# (*Z*)-*N*′-(4-Hydr­oxy-4-methyl­pentan-2-yl­idene)-2-(8-quinol­yloxy)acetohydrazide

**DOI:** 10.1107/S1600536809024957

**Published:** 2009-07-04

**Authors:** Li-Zi Yin, De-Guang Song, Song-Cai Liu

**Affiliations:** a College of Animal Science and Veterinary Medicine, Jilin University, Changchun 130062, People’s Republic of China

## Abstract

The title compound, C_17_H_21_N_3_O_3_, has a *Z* configuration about the N=N double bond. The molecular conformation is stabilized by intramolecular N—H⋯O and O—H⋯N hydrogen bonds.

## Related literature

For the potential pharmacological and anti­tumor properties of acidamide compounds, see: Harrop *et al.* (2003 ▶); Ren *et al.* (2002 ▶). For related structures, see: Lei *et al.* (2008 ▶); Yang *et al.* (2007 ▶).
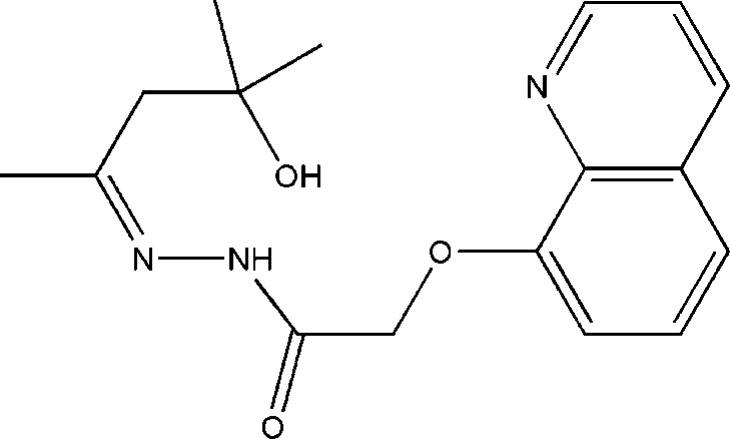


## Experimental

### 

#### Crystal data

C_17_H_21_N_3_O_3_
*M* *_r_* = 315.37
Orthorhombic, 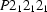

*a* = 9.3297 (12) Å
*b* = 10.1621 (13) Å
*c* = 18.213 (2) Å
*V* = 1726.7 (4) Å^3^
*Z* = 4
Mo *K*α radiation
μ = 0.09 mm^−1^
*T* = 273 K
0.20 × 0.18 × 0.15 mm

#### Data collection

Rigaku Saturn 724+ CCD detector diffractometer
Absorption correction: multi-scan (*CrystalClear*; Rigaku, 2000 ▶) *T*_min_ = 0.983, *T*_max_ = 0.987
9084 measured reflections
1761 independent reflections
1593 reflections with *I* > 2σ(*I*)
*R*_int_ = 0.025

#### Refinement

*R*[*F* ^2^ > 2σ(*F* ^2^)] = 0.035
*wR*(*F* ^2^) = 0.101
*S* = 1.04
1761 reflections
212 parameters
H-atom parameters constrained
Δρ_max_ = 0.15 e Å^−3^
Δρ_min_ = −0.16 e Å^−3^

### 

Data collection: *CrystalClear* (Rigaku, 2000 ▶); cell refinement: *CrystalClear*; data reduction: *CrystalClear*; program(s) used to solve structure: *SHELXS97* (Sheldrick, 2008 ▶); program(s) used to refine structure: *SHELXL97* (Sheldrick, 2008 ▶); molecular graphics: *SHELXTL* (Sheldrick, 2008 ▶); software used to prepare material for publication: *SHELXTL*.

## Supplementary Material

Crystal structure: contains datablocks global, I. DOI: 10.1107/S1600536809024957/fj2222sup1.cif

Structure factors: contains datablocks I. DOI: 10.1107/S1600536809024957/fj2222Isup2.hkl

Additional supplementary materials:  crystallographic information; 3D view; checkCIF report

## Figures and Tables

**Table 1:** Hydrogen-bond geometry (Å, °)

*D*—H⋯*A*	*D*—H	H⋯*A*	*D*⋯*A*	*D*—H⋯*A*
O3—H3⋯N1	0.82	2.10	2.820 (3)	146
N2—H2⋯O3	0.86	1.97	2.753 (2)	151

**Fig. 1.**
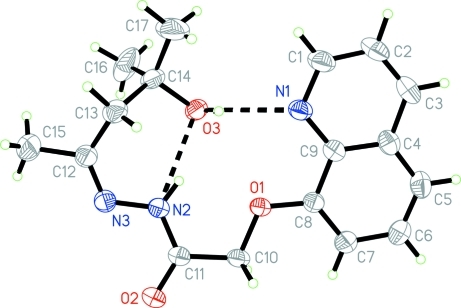
The molecular structure of (I). Displacement ellipsoids are drawn at the 30% probability level and H atoms are shown as small spheres. The O—H···N and N—H···O intramolecular hydrogen bond are shown dashed.

**Fig. 2.**
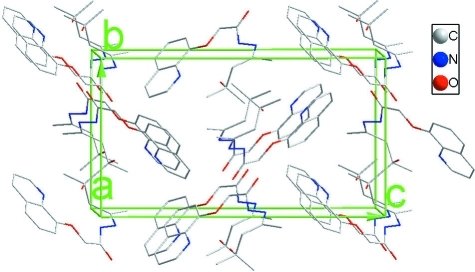
The crystal packing for (I).

## supplementary crystallographic information

### Comment

Acidamide compounds have been found to possess potential pharmacological and antitumor properties (Harrop *et al.*,2003; Ren *et al.*,2002). Up to now, a scant few of Acidamide compounds have been appeared (Lei *et al.*,2008; Yang *et al.*,2007). As a further study of such compounds, we report here the structure of the title compound.

In the molecule of (I) (Fig. 1), the bond lengths and angles are within normal ranges. In the crystal structure, intramolecular O—H···N and N—H···O hydrogen bonds (Table 1) seem to be effective in the stabilization of the structure.

### Experimental

3-hydroxy-3-methylbutanal (0.1 mmol, 10.2 mg) and 2-(quinolin-8-yloxy)acetohydrazide (0.1 mmol, 21.7 mg) were dissolved in methanol(20 ml). Then the mixture was stirred and refluxed for 1 h, and cooled to room temperature. After keeping the solution in air for about two weeks, yellow block crystals of the title compound were abtained. yield: 60% (based on 2-(quinolin-8-yloxy)acetohydrazide). Anal calcd for C_17_H_21_N_3_O_3_: C, 64.74%; H, 6.71%; N, 13.32%. Found: C, 64.46%; H, 6.48%; N, 13.59%.

### Refinement

H atoms of OH and NH groups were located in difference syntheses and constrained to ride on its parent atom [O—H = 0.82 Å and *U*_iso_(H) = 1.5*U*_eq_(O) (for OH); N—H = 0.86 Å and *U*_iso_(H) = 1.2*U*_eq_(N) (for NH)]. The remaining H atoms were positioned geometrically, with C—H = 0.93, 0.97 and 0.96 Å for aromatic, methylene and methyl H atoms, respectively, and constrained to ride on their parent atoms, with *U*_iso_(H) = *xU*_eq_(C), where *x* = 1.2 for aromatic and methylene H atoms, and *x* = 1.5 for methyl H atoms.

Friedel data were measured by MoKa radiation, but as there are no atoms heavier than Si, the absolute structure cannot been determined reliably and Friedel-pair data were averaged.

### Crystal data

**Table:**

C_17_H_21_N_3_O_3_	*F*(000) = 672
*M**_r_* = 315.37	*D*_x_ = 1.213 Mg m^−^^3^
Orthorhombic, *P*2_1_2_1_2_1_	Mo *K*α radiation, λ = 0.71073 Å
Hall symbol: P 2ac 2ab	Cell parameters from 4517 reflections
*a* = 9.3297 (12) Å	θ = 2.2–24.9°
*b* = 10.1621 (13) Å	µ = 0.09 mm^−^^1^
*c* = 18.213 (2) Å	*T* = 273 K
*V* = 1726.7 (4) Å^3^	Block, colorless
*Z* = 4	0.20 × 0.18 × 0.15 mm

### Data collection

**Table:**

Rigaku Saturn 724+ CCD detector diffractometer	1761 independent reflections
Radiation source: fine-focus sealed tube	1593 reflections with *I* > 2σ(*I*)
graphite	*R*_int_ = 0.025
Detector resolution: 9 pixels mm^-1^	θ_max_ = 25.0°, θ_min_ = 2.2°
ω scans	*h* = −11→10
Absorption correction: multi-scan (*CrystalClear*; Rigaku, 2000)	*k* = −12→11
*T*_min_ = 0.983, *T*_max_ = 0.987	*l* = −20→21
9084 measured reflections	

### Refinement

**Table:**

Refinement on *F*^2^	Primary atom site location: structure-invariant direct methods
Least-squares matrix: full	Secondary atom site location: difference Fourier map
*R*[*F*^2^ > 2σ(*F*^2^)] = 0.035	Hydrogen site location: inferred from neighbouring sites
*wR*(*F*^2^) = 0.101	H-atom parameters constrained
*S* = 1.04	*w* = 1/[σ^2^(*F*_o_^2^) + (0.0681*P*)^2^ + 0.1302*P*] where *P* = (*F*_o_^2^ + 2*F*_c_^2^)/3
1761 reflections	(Δ/σ)_max_ = 0.001
212 parameters	Δρ_max_ = 0.15 e Å^−^^3^
0 restraints	Δρ_min_ = −0.16 e Å^−^^3^

### Special details

**Table:**

Geometry. All e.s.d.'s (except the e.s.d. in the dihedral angle between two l.s. planes) are estimated using the full covariance matrix. The cell e.s.d.'s are taken into account individually in the estimation of e.s.d.'s in distances, angles and torsion angles; correlations between e.s.d.'s in cell parameters are only used when they are defined by crystal symmetry. An approximate (isotropic) treatment of cell e.s.d.'s is used for estimating e.s.d.'s involving l.s. planes.
Refinement. Refinement of *F*^2^ against ALL reflections. The weighted *R*-factor *wR* and goodness of fit *S* are based on *F*^2^, conventional *R*-factors *R* are based on *F*, with *F* set to zero for negative *F*^2^. The threshold expression of *F*^2^ > σ(*F*^2^) is used only for calculating *R*-factors(gt) *etc*. and is not relevant to the choice of reflections for refinement. *R*-factors based on *F*^2^ are statistically about twice as large as those based on *F*, and *R*- factors based on ALL data will be even larger.

### Fractional atomic coordinates and isotropic or equivalent isotropic displacement parameters (Å^2^)

**Table:**

	*x*	*y*	*z*	*U*_iso_*/*U*_eq_	
O1	−0.14080 (16)	−0.04549 (14)	0.89142 (8)	0.0517 (4)	
O2	−0.0517 (2)	−0.26572 (17)	1.03499 (9)	0.0689 (5)	
O3	0.06563 (17)	0.17486 (16)	0.93454 (10)	0.0610 (4)	
H3	0.0565	0.1430	0.8934	0.091*	
N1	−0.0639 (2)	0.1419 (2)	0.79583 (10)	0.0575 (5)	
N2	0.03984 (19)	−0.06920 (18)	0.99886 (10)	0.0520 (4)	
H2	0.0391	−0.0095	0.9654	0.062*	
N3	0.1400 (2)	−0.06223 (19)	1.05559 (10)	0.0570 (5)	
C1	−0.0301 (3)	0.2370 (3)	0.74871 (15)	0.0738 (7)	
H1	0.0575	0.2791	0.7551	0.089*	
C2	−0.1156 (4)	0.2780 (3)	0.69090 (14)	0.0781 (8)	
H2A	−0.0860	0.3458	0.6601	0.094*	
C3	−0.2431 (3)	0.2174 (3)	0.68019 (13)	0.0717 (8)	
H3A	−0.3014	0.2425	0.6412	0.086*	
C4	−0.2882 (3)	0.1155 (2)	0.72835 (12)	0.0578 (6)	
C5	−0.4203 (3)	0.0503 (3)	0.72093 (14)	0.0721 (7)	
H5	−0.4827	0.0729	0.6832	0.087*	
C6	−0.4560 (3)	−0.0455 (3)	0.76902 (15)	0.0750 (7)	
H6	−0.5429	−0.0892	0.7632	0.090*	
C7	−0.3655 (3)	−0.0816 (3)	0.82796 (13)	0.0628 (6)	
H7	−0.3934	−0.1469	0.8607	0.075*	
C8	−0.2363 (2)	−0.0194 (2)	0.83633 (11)	0.0478 (5)	
C9	−0.1937 (2)	0.0816 (2)	0.78632 (11)	0.0488 (5)	
C10	−0.1728 (2)	−0.1518 (2)	0.94005 (12)	0.0542 (5)	
H10A	−0.1828	−0.2326	0.9122	0.065*	
H10B	−0.2628	−0.1349	0.9649	0.065*	
C11	−0.0544 (2)	−0.1672 (2)	0.99604 (11)	0.0492 (5)	
C12	0.2509 (3)	0.0078 (2)	1.04208 (14)	0.0640 (6)	
C13	0.2906 (3)	0.0687 (3)	0.96876 (16)	0.0684 (7)	
H13A	0.2699	0.0051	0.9305	0.082*	
H13B	0.3932	0.0841	0.9684	0.082*	
C14	0.2151 (3)	0.1985 (3)	0.94902 (19)	0.0747 (8)	
C15	0.3571 (4)	0.0213 (3)	1.10411 (19)	0.1010 (12)	
H15A	0.4434	−0.0252	1.0920	0.151*	
H15B	0.3167	−0.0149	1.1482	0.151*	
H15C	0.3786	0.1127	1.1117	0.151*	
C16	0.2178 (4)	0.2961 (3)	1.0126 (3)	0.1322 (18)	
H16A	0.1606	0.2629	1.0523	0.198*	
H16B	0.1797	0.3791	0.9965	0.198*	
H16C	0.3147	0.3080	1.0290	0.198*	
C17	0.2846 (4)	0.2557 (5)	0.8806 (3)	0.156 (2)	
H17A	0.2352	0.3346	0.8665	0.234*	
H17B	0.2792	0.1927	0.8413	0.234*	
H17C	0.3832	0.2757	0.8906	0.234*	

### Atomic displacement parameters (Å^2^)

**Table:**

	*U*^11^	*U*^22^	*U*^33^	*U*^12^	*U*^13^	*U*^23^
O1	0.0499 (8)	0.0465 (8)	0.0587 (8)	−0.0073 (7)	−0.0036 (7)	0.0125 (7)
O2	0.0794 (11)	0.0569 (9)	0.0705 (10)	−0.0125 (9)	−0.0105 (9)	0.0225 (8)
O3	0.0504 (8)	0.0505 (9)	0.0819 (10)	−0.0052 (7)	−0.0098 (8)	0.0055 (8)
N1	0.0578 (11)	0.0573 (11)	0.0576 (10)	−0.0058 (10)	0.0067 (9)	0.0118 (9)
N2	0.0524 (10)	0.0476 (9)	0.0559 (9)	−0.0032 (8)	−0.0013 (8)	0.0081 (8)
N3	0.0623 (11)	0.0507 (10)	0.0580 (10)	−0.0003 (9)	−0.0067 (9)	−0.0015 (9)
C1	0.0736 (17)	0.0745 (17)	0.0733 (15)	−0.0074 (14)	0.0140 (14)	0.0225 (14)
C2	0.089 (2)	0.0799 (18)	0.0652 (15)	0.0027 (17)	0.0173 (15)	0.0288 (14)
C3	0.086 (2)	0.0785 (17)	0.0504 (12)	0.0184 (16)	0.0044 (13)	0.0145 (12)
C4	0.0641 (14)	0.0624 (14)	0.0468 (11)	0.0124 (11)	0.0033 (10)	−0.0003 (10)
C5	0.0668 (15)	0.0862 (19)	0.0634 (13)	0.0068 (15)	−0.0140 (12)	0.0047 (15)
C6	0.0607 (14)	0.0828 (18)	0.0814 (16)	−0.0112 (14)	−0.0151 (13)	0.0023 (15)
C7	0.0557 (14)	0.0650 (14)	0.0677 (13)	−0.0069 (12)	−0.0026 (11)	0.0073 (12)
C8	0.0501 (11)	0.0445 (10)	0.0487 (10)	0.0018 (9)	0.0024 (9)	−0.0001 (8)
C9	0.0541 (12)	0.0448 (10)	0.0475 (10)	0.0065 (10)	0.0069 (9)	−0.0008 (9)
C10	0.0549 (12)	0.0460 (11)	0.0616 (12)	−0.0079 (10)	0.0025 (10)	0.0130 (10)
C11	0.0518 (11)	0.0444 (11)	0.0513 (10)	−0.0004 (10)	0.0060 (9)	0.0059 (9)
C12	0.0616 (13)	0.0463 (11)	0.0839 (15)	0.0000 (11)	−0.0131 (13)	0.0044 (11)
C13	0.0457 (12)	0.0603 (13)	0.0992 (17)	−0.0002 (11)	−0.0014 (13)	0.0088 (14)
C14	0.0490 (13)	0.0541 (13)	0.121 (2)	−0.0120 (11)	−0.0157 (15)	0.0222 (15)
C15	0.105 (3)	0.0767 (19)	0.121 (2)	−0.0196 (19)	−0.051 (2)	0.0065 (19)
C16	0.100 (3)	0.0574 (17)	0.239 (5)	0.0046 (17)	−0.081 (3)	−0.032 (2)
C17	0.064 (2)	0.174 (4)	0.231 (5)	−0.028 (2)	−0.006 (3)	0.137 (4)

### Geometric parameters (Å, °)

**Table:**

O1—C8	1.368 (2)	C7—C8	1.370 (3)
O1—C10	1.429 (2)	C7—H7	0.9300
O2—C11	1.228 (3)	C8—C9	1.429 (3)
O3—C14	1.440 (3)	C10—C11	1.512 (3)
O3—H3	0.8200	C10—H10A	0.9700
N1—C1	1.331 (3)	C10—H10B	0.9700
N1—C9	1.368 (3)	C12—C15	1.508 (4)
N2—C11	1.329 (3)	C12—C13	1.518 (4)
N2—N3	1.395 (3)	C13—C14	1.538 (4)
N2—H2	0.8600	C13—H13A	0.9700
N3—C12	1.280 (3)	C13—H13B	0.9700
C1—C2	1.385 (4)	C14—C17	1.520 (5)
C1—H1	0.9300	C14—C16	1.525 (5)
C2—C3	1.354 (4)	C15—H15A	0.9600
C2—H2A	0.9300	C15—H15B	0.9600
C3—C4	1.421 (4)	C15—H15C	0.9600
C3—H3A	0.9300	C16—H16A	0.9600
C4—C5	1.406 (4)	C16—H16B	0.9600
C4—C9	1.418 (3)	C16—H16C	0.9600
C5—C6	1.351 (4)	C17—H17A	0.9600
C5—H5	0.9300	C17—H17B	0.9600
C6—C7	1.414 (4)	C17—H17C	0.9600
C6—H6	0.9300		
			
C8—O1—C10	117.74 (16)	C11—C10—H10B	109.6
C14—O3—H3	109.5	H10A—C10—H10B	108.2
C1—N1—C9	117.0 (2)	O2—C11—N2	125.1 (2)
C11—N2—N3	120.64 (17)	O2—C11—C10	119.26 (19)
C11—N2—H2	119.7	N2—C11—C10	115.61 (17)
N3—N2—H2	119.7	N3—C12—C15	115.9 (2)
C12—N3—N2	115.31 (18)	N3—C12—C13	126.4 (2)
N1—C1—C2	124.9 (3)	C15—C12—C13	117.5 (2)
N1—C1—H1	117.5	C12—C13—C14	116.3 (2)
C2—C1—H1	117.5	C12—C13—H13A	108.2
C3—C2—C1	118.6 (3)	C14—C13—H13A	108.2
C3—C2—H2A	120.7	C12—C13—H13B	108.2
C1—C2—H2A	120.7	C14—C13—H13B	108.2
C2—C3—C4	120.2 (2)	H13A—C13—H13B	107.4
C2—C3—H3A	119.9	O3—C14—C17	109.0 (3)
C4—C3—H3A	119.9	O3—C14—C16	105.3 (3)
C5—C4—C9	120.2 (2)	C17—C14—C16	111.5 (3)
C5—C4—C3	122.9 (2)	O3—C14—C13	110.1 (2)
C9—C4—C3	116.9 (2)	C17—C14—C13	108.9 (3)
C6—C5—C4	119.6 (2)	C16—C14—C13	111.9 (3)
C6—C5—H5	120.2	C12—C15—H15A	109.5
C4—C5—H5	120.2	C12—C15—H15B	109.5
C5—C6—C7	122.1 (3)	H15A—C15—H15B	109.5
C5—C6—H6	119.0	C12—C15—H15C	109.5
C7—C6—H6	119.0	H15A—C15—H15C	109.5
C8—C7—C6	119.4 (2)	H15B—C15—H15C	109.5
C8—C7—H7	120.3	C14—C16—H16A	109.5
C6—C7—H7	120.3	C14—C16—H16B	109.5
O1—C8—C7	124.42 (19)	H16A—C16—H16B	109.5
O1—C8—C9	115.21 (18)	C14—C16—H16C	109.5
C7—C8—C9	120.4 (2)	H16A—C16—H16C	109.5
N1—C9—C4	122.4 (2)	H16B—C16—H16C	109.5
N1—C9—C8	119.15 (19)	C14—C17—H17A	109.5
C4—C9—C8	118.4 (2)	C14—C17—H17B	109.5
O1—C10—C11	110.08 (17)	H17A—C17—H17B	109.5
O1—C10—H10A	109.6	C14—C17—H17C	109.5
C11—C10—H10A	109.6	H17A—C17—H17C	109.5
O1—C10—H10B	109.6	H17B—C17—H17C	109.5
			
C11—N2—N3—C12	159.0 (2)	C5—C4—C9—C8	−0.1 (3)
C9—N1—C1—C2	−0.6 (4)	C3—C4—C9—C8	179.3 (2)
N1—C1—C2—C3	−0.5 (5)	O1—C8—C9—N1	1.0 (3)
C1—C2—C3—C4	1.0 (4)	C7—C8—C9—N1	−179.8 (2)
C2—C3—C4—C5	178.9 (3)	O1—C8—C9—C4	−179.09 (18)
C2—C3—C4—C9	−0.4 (3)	C7—C8—C9—C4	0.1 (3)
C9—C4—C5—C6	−0.5 (4)	C8—O1—C10—C11	178.94 (17)
C3—C4—C5—C6	−179.8 (3)	N3—N2—C11—O2	−9.8 (3)
C4—C5—C6—C7	1.1 (4)	N3—N2—C11—C10	169.88 (18)
C5—C6—C7—C8	−1.1 (4)	O1—C10—C11—O2	−168.6 (2)
C10—O1—C8—C7	5.2 (3)	O1—C10—C11—N2	11.7 (3)
C10—O1—C8—C9	−175.65 (17)	N2—N3—C12—C15	177.3 (2)
C6—C7—C8—O1	179.5 (2)	N2—N3—C12—C13	−7.7 (3)
C6—C7—C8—C9	0.4 (3)	N3—C12—C13—C14	81.1 (3)
C1—N1—C9—C4	1.3 (3)	C15—C12—C13—C14	−104.0 (3)
C1—N1—C9—C8	−178.8 (2)	C12—C13—C14—O3	−69.5 (3)
C5—C4—C9—N1	179.8 (2)	C12—C13—C14—C17	171.0 (3)
C3—C4—C9—N1	−0.8 (3)	C12—C13—C14—C16	47.2 (3)

### Hydrogen-bond geometry (Å, °)

**Table:**

*D*—H···*A*	*D*—H	H···*A*	*D*···*A*	*D*—H···*A*
O3—H3···N1	0.82	2.10	2.820 (3)	146
N2—H2···O3	0.86	1.97	2.753 (2)	151
